# Health – Nutrition- Fitness- Wellness- Present and Future

**Published:** 2012

**Authors:** Victor Lorin Purcarea

**Affiliations:** ”Carol Davila” University of Medicine and Pharmacy, Bucharest

The second edition of the **International Congress Health-Nutrition-Fitness-Wellbeing – SANABUNAINT 2012** took place in Falticeni, Suceava, during 19-21 of October 2012.

**Fig. 1:**
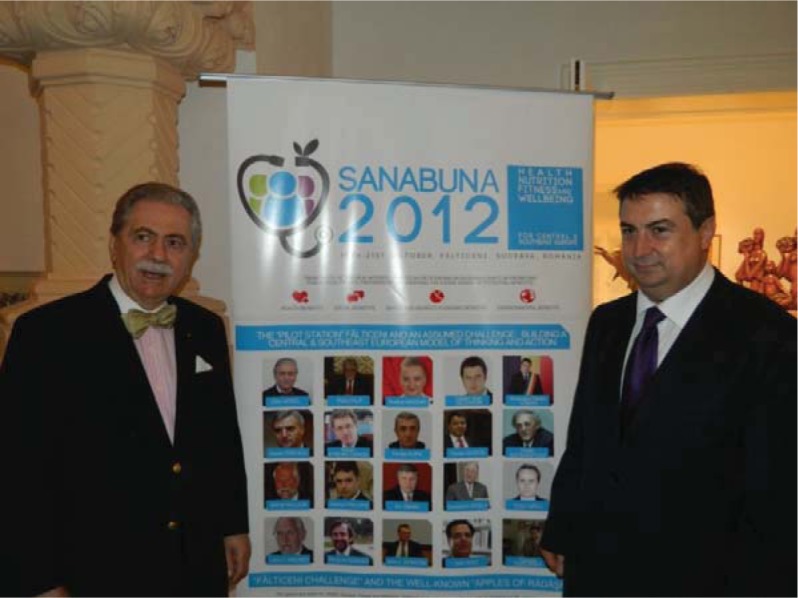
Prof. Eliot Sorel – the representative of GWU School of Medicine and the School of Public Health in Washington D.C. and Prof. Theodor Valentin Purcărea – President of the Romanian Committee of Distribution, member of the Board of the International Association of Distribution – A.I.D.A. - Bruxelles

The event, which was organized under the patronage of the Romanian Patriarchy, the Romanian Academy, the Ministry of Public Health, the Ministry of Agriculture and Rural Development, the Ministry of Education, Research, Youth and Sport, has benefited from the presence of some important personalities such as: Prof. Eliot Sorel, Representative of GWU School of Medicine and the School of Public Health in Washington D.C.; Acad. Ion Ababii, Rector of “Nicolae Testemitianu” University of Medicine and Pharmacy in Chişinău; Prof. Florian Popa, President of the Congress; Petru Filip, Vice-president of the Senate of Romania; Rodica Nassar, President of the Health Commission of the Chamber of Deputies, Romanian Parliament; Florian Bodoc, Secretary of State in the Ministry of Health; Prof. Vasile Astărăstoae, President of the Romanian College of Physicians; Florin Sinescu, Prefect of Suceava; Ioan Cătălin Nechifor, President of Suceava Town Council; Cristian Adomniţei, President of Iasi Town Council; Gheorghe Cătălin Coman, Mayor of Falticeni Town; Prof. Theodor Valentin Purcărea, President of the Romanian Committee of Distribution, member of the Board of the International Association of Distribution – A.I.D.A. – Bruxelles; Prof. Remus Pricopie, Rector of the National School of Political and Administrative Studies. An impressive number of physicians took part in the event, many of whom being youngsters or even students.

**Fig. 2:**
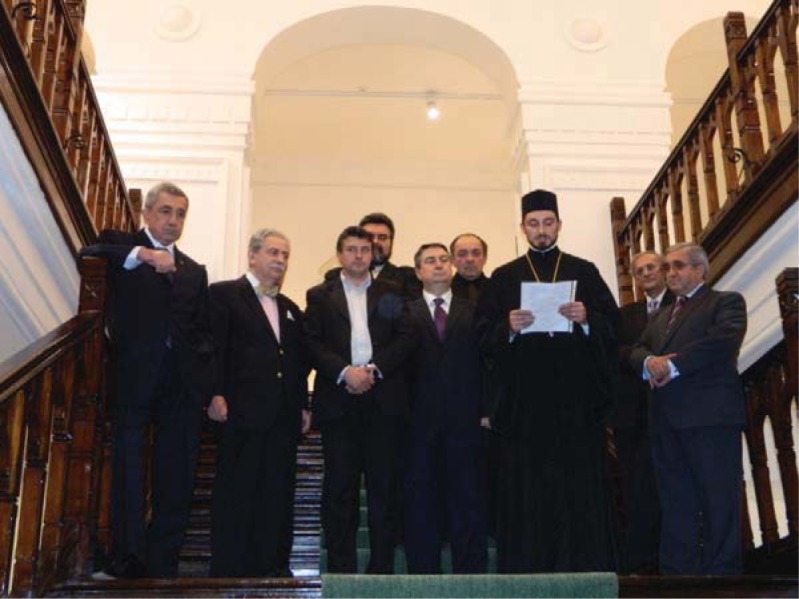
The Official Opening of the Congress. The message of H.B. Patriarch Daniel of the Romanian Orthodox Church addressing the participants at the Congress

The Congress was held in the generous space of “Ion Irimescu” Art Museum and was opened by the personal representative of H.B. Patriarch Daniel of the Romanian Orthodox Church, who transmitted the message addressed to the participants. The festive opening was emotional due to the “Choir of Students in Medicine and Pharmacy” at “Carol Davila” University of Medicine and Pharmacy in Bucharest and the “Falticeni Choir”, who have created delightful moments for the audience through the performance in the concert.

**Fig. 3:**
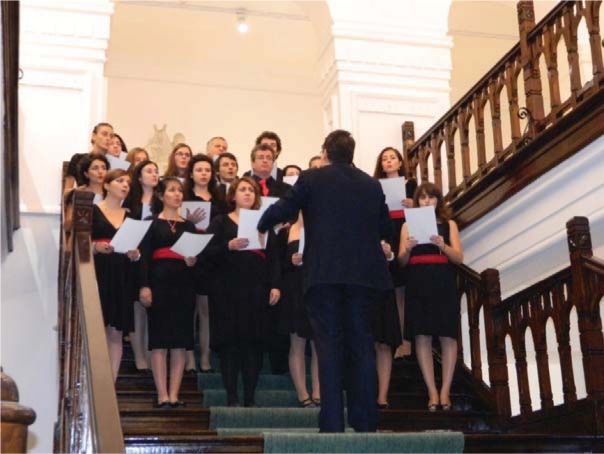
Choir of Students in Medicine and Pharmacy at “Carol Davila” University of Medicine and Pharmacy in Bucharest

The event, which took place in the context of the 9th edition of the **Apple Festival**– “The Apples of Rădăşeni”, and as an expression of the responsible preoccupation of the **Falticeni Town Hall** and **Suceava Town Council** in promoting the Public-Civic-Private Partnership, while considering the entire series of potential benefits (health, social, direct and indirect economical, environmental), under the slogan **“*Pilot Station*” *Falticeni and an assumed challenge: Constructing a Central Regional and Eastern-European Model of Thinking and Acting*”**, was held for two days and has brought into light discussion themes such as the following: The Danube Delta – TD Education Research Innovation Development-*Eliot Sorel*; Communication and Information Technology: Protecting, Promoting Health and Preventing Diseases – *Remus Pricopie*; Health-Nutrition-Fitness-Wellness – an actual challenge- *Theodor Valentin Purcărea*; Impact of the metabolic affections on the affective disorders- *Maria Ladea, Cristina Maria Barbu, Dan Petru Roşu*; Digestive infections with Salmonella enterica, the subspecies enterica resistant to antibodies – a problem of public health – *Victor Lorin Purcărea, Bogdan-Ioan Coculescu, Elena Pahonţu*; The combination of enteral nutrition with the parenteral one postoperatively – *Mihai Dobra, Mihaela Coman, Maria Tomescu*; The influences of healthy nutrition on the harmonious psychical development of the youngsters – *Simona Vlădică*; Nutrition principles in pregnant women with diabetes – *Antoine Edu, Alexandru Matei, Radu Mateescu*, etc.

**Fig. 4:**
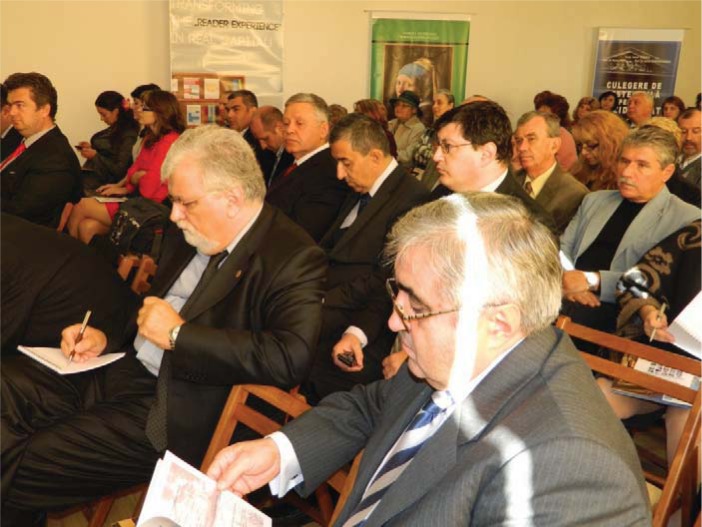
The Congress has been attended with interest by the numerous participants

The program was completed by a section of round tables on themes such as: A trip in the history and culture of Fălticeni Town and Innovative community approaches regarding the healthy lifestyle in children and adolescents.

**Fig. 5:**
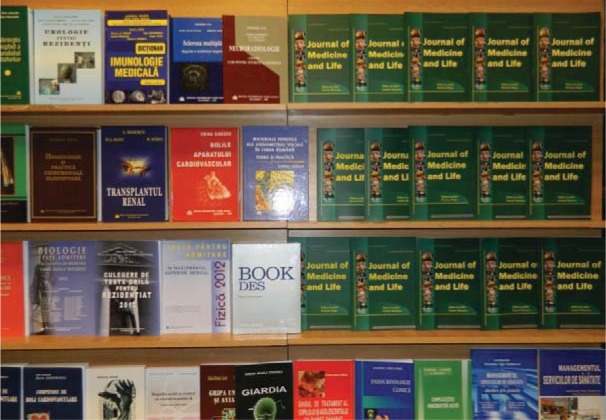
One of the stands of “Carol Davila” University Press, “Carol Davila” University of Medicine and Pharmacy

On this occasion, “Carol Davila” University Press has presented a book exhibition, which was very well received both by the specialists and the general public. Among the book presentations that took place are also the following: “Surgery and Orthopedic Pediatrics”; “Nutrition and Mental Health”; “Larynx cancer – anatomo-surgical correlations”; “The infection with hepatitis B and D”; “Practical guide of bilateral ocular ultrasound”; “21st century – Global Mental Heart”.

The participation in the manifestations incurred by the “Apple Festival” in the Central Market of Falticeni Town took place during the Congress.

**Fig. 6:**
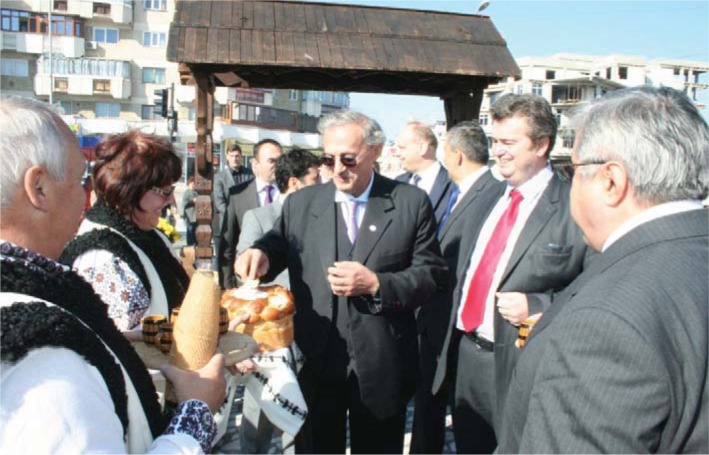
The traditional wellcome with “bread and salt” to the “Apple Festival” in the Central Market

The image presents Prof. Vasile Astărăstoae, MD – President of the Romanian College of Physicians, the Rector of “Gr. T. Popa” University of Medicine and Pharmacy in Iaşi and Ioan Cătălin Nechifor President of Suceava Town Council

**Fig. 7:**
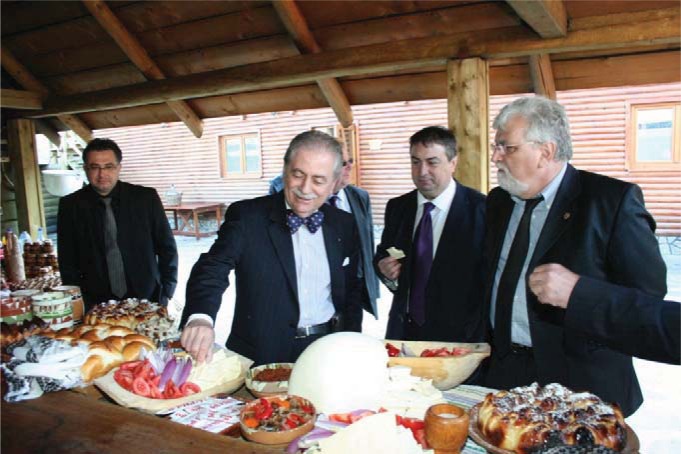
Tasting the bio products: Prof. Eliot Sorel, MD; Prof. Dr. Theodor Valentin Purcărea and Prof. Dr. Petru Filip – Vice-president of the Senate of Romania

The Congress was closed with the awards ceremony for the best papers and a magnificent show of “Ciprian Porumbescu” folkloric group.

**Fig. 8:**
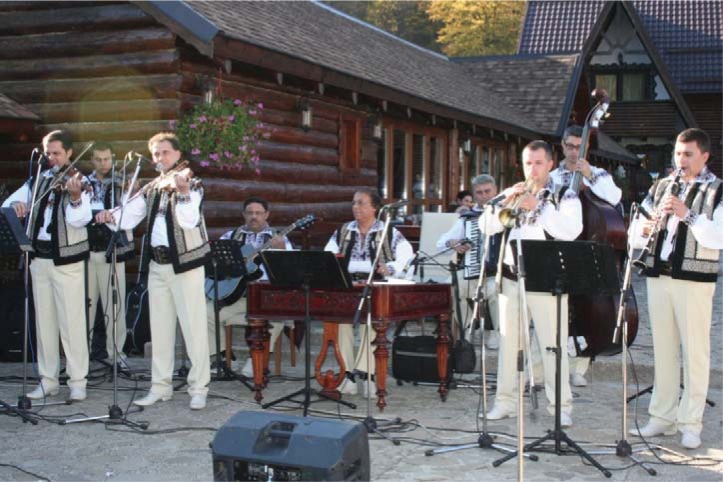
The Instrumental Band of “Ciprian Porumbescu” Folkloric Band in Suceava

